# Supplementary material for: Identification of photoperiod-induced specific miRNAs in the adrenal glands of Sunite sheep (Ovis aries)
Source: Front Vet Sci. 2022 Jul 22;9:888207. doi: 10.3389/fvets.2022.888207 (PMC9354845; doi:10.3389/fvets.2022.888207)
Supplement: Supplementary file 1 [file Data_Sheet_1.ZIP › Supplementary file/miRDeep2 file.zip!/miRDeep2 file/Novel_Structure/SP42A2/Novel_144.pdf]

Provisional ID	:	NC_019460.2_8439
Score total	:	2.6
Score for star read(s)	:	-1.3
Score for read counts	:	0
Score for mfe	:	2.3
Score for randfold	:	1.6
Score for cons. seed	:	
Total read count	:	76
Mature read count	:	73
Loop read count	:	0
Star read count	:	3

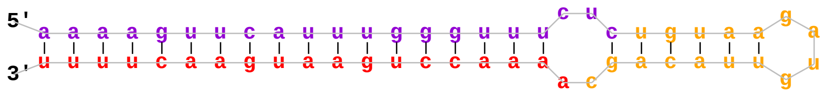

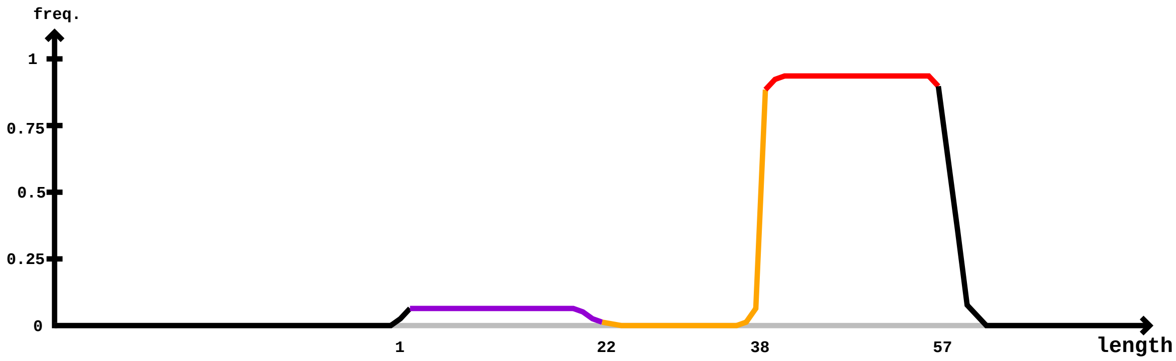

Star Mature

```
5'- cacugaggggagucacauaguauuggacuggccaaaaaaguucauuuggguuucucuguaagauguuacagcaaaaccugaaugaacuuuuuuggccaacccaauauuuguu  -3'  obs
    cacugaggggagucacauaguauuggacuggccaaaaaaguucauuuggguuucucuguaagauguuacagcaaaaccugaaugaacuuuuuuggccaacccaauauuuguu       exp
    .(((......))).(((.((((((((..(((((((((((((((((((((((((..((((((....))))))..)))))))))))))))))))))))))..))))))))))).   reads   mm   sample
    ..................................aaaaaguucGuuuggguuuc..........................................................   2   1   seq
    ...................................Uaaaguucauuuggguuu...........................................................   1   1   seq
    ...................................aaaaguucGuuuggguuucu.........................................................   1   1   seq
    ...................................aaaaguucauuuggguuuUc.........................................................   1   1   seq
    ......................................................................gcaaaaccugaaugaacuuuuu....................   1   0   seq
    .......................................................................Uaaaaccugaaugaacuuu......................   1   1   seq
    .......................................................................caaaaccugaaugaacuuuuu....................   2   0   seq
    .......................................................................Uaaaaccugaaugaacuuuuu....................   1   1   seq
    ........................................................................aaaaccAgaaugaacuuu......................   2   1   seq
    ........................................................................aaaaUcugaaugaacuuuu.....................   23  1   seq
    ........................................................................aaaaccugaaugaacCuuu.....................   1   1   seq
    ........................................................................Gaaaccugaaugaacuuuuu....................   2   1   seq
    ........................................................................aaaCccugaaugaacuuuuu....................   1   1   seq
    ........................................................................aaaaccAgaaugaacuuuuu....................   9   1   seq
    ........................................................................aaaaccugaaugaacuuuuC....................   1   1   seq
    ........................................................................aaaaUcugaaugaacuuuuuu...................   16  1   seq
    ........................................................................aaaaccugaaugaacuuuuuu...................   3   0   seq
    ........................................................................aaaaccugaaugaacuuuuuug..................   1   0   seq
    ........................................................................aaaaUcugaaugaacuuuuuug..................   2   1   seq
    ........................................................................aaaaccugaaugaacuuuuuugg.................   2   0   seq
    ........................................................................aaaaccugaaugaacuuuuuGgg.................   1   1   seq
    .........................................................................aaaccugaaugaacuuuuu....................   1   0   seq
    .........................................................................aaaccCgaaugaacuuuuuu...................   2   1   seq
    ..........................................................................aaccCgaaugaacuuuuuu...................   1   1   seq
```
